# Comparing Airway Analysis in Two-Time Points after Rapid Palatal Expansion: A CBCT Study

**DOI:** 10.3390/jcm12144686

**Published:** 2023-07-14

**Authors:** Ioannis A. Tsolakis, Olga-Elpis Kolokitha

**Affiliations:** 1 Department of Orthodontics, Faculty of Health Sciences, School of Dentistry, Aristotle University of Thessaloniki, 54124 Thessaloniki, Greece; okolok@dent.auth.gr; 2 Department of Orthodontics, School of Dental Medicine, Case Western Reserve University, Cleveland, OH 44106, USA

**Keywords:** rapid palatal expansion, nasal airway, airway volume, airway minimal cross-sectional area

## Abstract

Background: The aim of this study is to investigate the upper airway analysis at two-time points after the rapid maxillary expansion was performed, using cone-beam computed tomography. Methods: Subjects from the Orthodontic Department at the Aristotle University of Thessaloniki with unilateral or bilateral posterior crossbite were screened according to the selection criteria. A sample size calculation was performed, and a total of 14 subjects were recruited. All subjects received a rapid maxillary expansion with a Hyrax-type device as part of their comprehensive treatment. A CBCT was taken before the treatment (T1), immediately after the expansion was completed (T2), and 6 months after (T3). Their upper airway was measured using the CBCT images. Airway volume (V) and minimal cross-sectional area (MCS) were extracted and compared using SPSS to analyze the means. Results: A statistically significant difference was found between all time points regarding both V and MCS (*p* < 0.001, *p* = 0.001). There was a statistically significant increase in both V and MCS measurements immediately after RPE expansion (T1-T2) and six months after expansion (T1-T3). Between the end of expansion and 6 months after (T2-T3), there was a decrease in V and no statistical difference in MCS. Conclusions: RPE can significantly increase the volume and minimal cross-sectional area of the nasal passage airway.

## 1. Introduction

In order to facilitate normal craniofacial development, early identification and treatment are preferred for dentofacial abnormalities that arise as a result of upper airway constriction [1]. Obstructive sleep apnea (OSA), sleep disordered breathing, and upper airway morphology have all been researched more recently, and there is a general consensus that early care of these problems may improve patients’ long-term medical and dental outcomes [2]. More than 20% of teenagers snore at least a few times each month, 6% snore constantly or almost constantly, and 2.5% to 6.1% of teenagers experience apnea-like symptoms [3,4]. Orthodontists use the term “maxillary constriction” to characterize a maxilla that is narrow in the lateral dimension in comparison to other facial bones, particularly the mandible. Subjects with maxillary constriction are known to have higher nasal resistance and struggle to breathe through their nose [5,6], characteristics that are frequently seen in OSA patients. Low tongue position is also linked to maxillary constriction [7], that may lead to retroglossal airway narrowing, which is another OSA symptom [8,9].

A well-established orthodontic treatment method to address transverse maxillary constriction is rapid palatal expansion (RPE). It was first described by Angell in the 19th century and mentioned for its effects on the maxilla, as well as being recommended as a treatment for respiratory disorders [10]. According to the RPE technique, strong strains produced by RPE appliances lead to the expansion of the midpalatal suture and an increase in the transverse widths of the maxilla [11,12,13]. In addition to expanding the nasal cavity, this tension causes the separation of the two maxillary halves from the midpalatal suture. The impacts of RPE might affect the morphology of the nasal cavity and, consequently, the advancement of the nasal cavity’s dimensions since the maxilla is so close to the nasal floor [12,13,14,15,16,17,18]. An increase in nasal volume and maybe a reduction in nasal resistance as well as an improvement in the nasal airway are produced by the enlargement of the nasal cavity. The Angell method was tried several times over the years with varied degrees of success, and it was finally revived by Haas [19,20], who claimed that using RPE caused significant modifications in the nasomaxillary complex. Wertz [21] added that maxillary suture opening might be beneficial for patients who had nasal stenosis in the anteroinferior region of their nasal chambers. Another negative consequence of maxillary constriction is its role in the pathophysiology of obstructive sleep apnea, where the retro-position of the tongue may result in a restricted oropharyngeal airway [22,23]. Additionally, RME is known to alter the mandibular posture, which may alter the volume and size of the oropharyngeal (OP) airway [24].

Multiple methods, including lateral and anteroposterior radiographs, acoustic rhinometry approaches, and multislice computed tomography, have been used to study the effects of RPE on nasal size and volume [25,26,27,28,29,30,31,32,33,34]. Cone beam computed tomography (CBCT) and accompanying software have a recently advanced method that gives the clinician the ability to reconstruct and measure the upper airway as a solid structure. The advantages of CBCT technology over other airway assessment techniques are reduced costs, less radiation exposure, quicker scanning times, and overall accuracy [35,36,37,38,39]. In 2016, Tsolakis et al. compared the CBCT method to acoustic reflection methods for the measurement of the upper airway volume and minimal cross-sectional area, and they found that CBCT is as accurate as the acoustic reflection methods. The results of this study proved that CBCT is an accurate method for measuring upper airway volume and minimal cross-sectional area [40]. This technique has also been utilized in studies to assess how RPE affects the nasal airway [3,41,42,43]. According to the present limited literature, artificial intelligence gives clinicians the ability to measure the airway volume and minimal cross-sectional area accurately from CBCT pictures [44].

Improvements in respiratory function may not necessarily translate into changes in the nasal cavity’s shape. The relationship between the nasal cavity and respiratory function has been the subject of numerous research in the literature; however, the association between the two is debatable [3,30,45]. Various research that studied the effects of maxillary expansion on the airway have found that the expansion of the nasal width and volume may lead to a reduction in nasal resistance [46,47]. Multiple studies have discovered varied degrees of expanded nasal cavity dimensions and decreased nasal obstruction in association with RPE. Despite the current growth in interest in this subject, additional research is still needed to strengthen the conclusions. Furthermore, to our knowledge, there is no study that looked over the airway volume and minimal cross-sectional area changes in the nasal passage immediately after the expansion and after the retention period. The aim of this prospective clinical trial was to investigate the changes on the airway volume and cross-sectional area in the nasal passage area in growing patients using CBCT at two-time points after rapid palatal expansion.

## 2. Materials and Methods

Prior to the start of this study, an institutional review board approval was obtained from the Ethical Committee of the School of Dentistry of the Aristotle University of Thessaloniki in Greece (Protocol No. 56/05-07-19). Subjects were recruited from the Orthodontic Department at Aristotle University of Thessaloniki, Greece. Inclusion criteria consisted of growing patients characterized with a unilateral or bilateral crossbite, and fully erupted first upper permanent molars who need RPE as part of their non-extraction orthodontic therapy. Patients who were non-growing or had craniofacial defects, such as cleft lip and palate, several missing or dysplastic teeth, patients who had at least one permanent tooth extracted, and patients who had previously undergone orthodontic treatment were excluded from this study. All parents and/or guardians of the patients who took part in the study signed an informed permission form. A sample size calculation was performed using data from previous studies, suggesting 14 subjects for a confidence level of 95%. A sample of 14 subjects (6 boys and 8 girls with a mean age of 10.82 and 11.34 years, respectively) was recruited and used in this project. All subjects received a rapid maxillary expansion as the initial part of their orthodontic treatment.

All patients had a Hyrax expander with bands placed on their first maxillary permanent molars and, first premolars, or maxillary deciduous first molars. The same lab fabricated all Hyrax expanders, and the same business produced all screws. The same laboratory fabricated all Hyrax expanders and all Hyrax’s screws were made by the same orthodontic manufacturer. The expansion screw was turned twice daily (0.25 mm per turn, 0.5 mm daily) until the mandibular first molar’s buccal cusp and the maxillary first molar’s palatal cusp occluded. The Hyrax screw was stabilized after expansion with ligature wire and light-cured composite.

**Figure 1:**
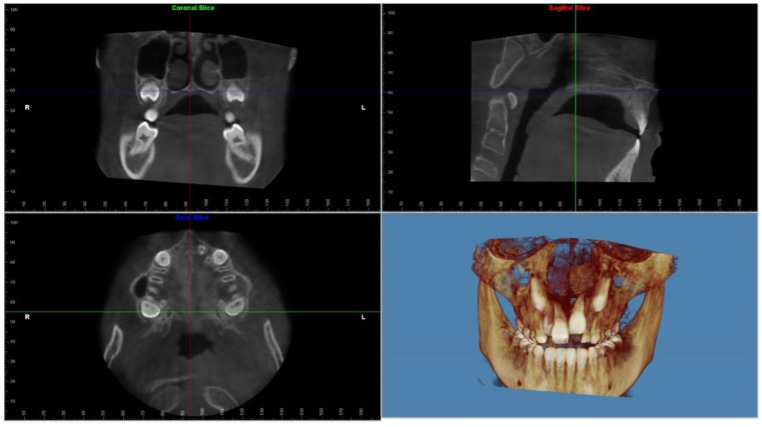
CBCT orientation: orient the subject in the coronal view so that the axial plane is aligned with the floor of the inferior turbinate, and in the axial view so that the coronal plane is aligned with the left and right greater foramen palatine. Orientation of the sagittal plane with mid-palate in the axial view and the axial plane with the palatal plane in the sagittal view.

**Figure 2:**
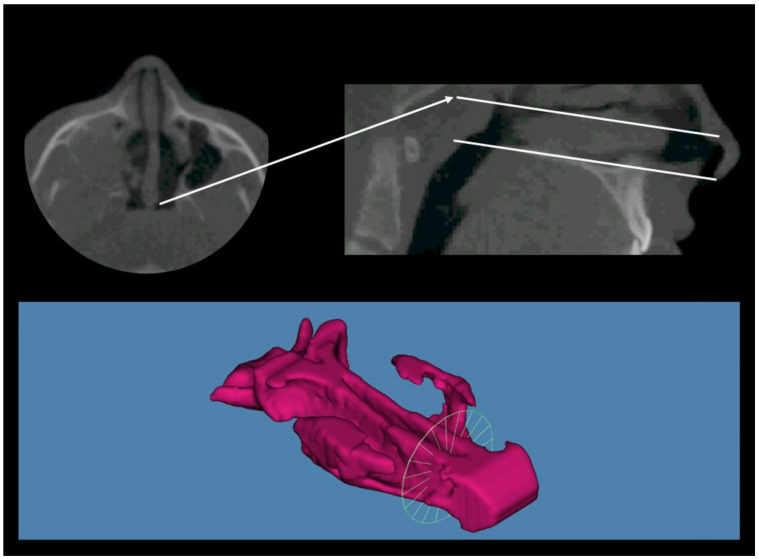
The borders of nasal passage (NP). Nasal passage area was defined as the area between the palatal plane and a parallel plane passing through the last axial slice before the nasal septum fused with the posterior pharyngeal wall. The volume and minimal cross-sectional area measurements are presented.

A CBCT was taken before the treatment (T1), immediately after the expansion was completed (T2), and 6 months after the expansion was completed allowing the ossification of the midpalatal suture (T3). A qualified technician operated the same scanner (Soredex Scanora 3D) for all radiography tests. The 3D scans were performed at 90 KV, 10 mA, and 0.35 mm voxel size with a large field of view (FOV: 75 mm height × 145 mm diameter). The CBCT data were exported as DICOM files. All CBCT images were imported into Dolphin 3D Imaging program version 11.95 SP3 (Dolphin Imaging and Management Solutions, Chatsworth, CA, USA), where they were oriented (Figure 1). Data collected from the CBCT image included total nasal volume, and nasal minimal cross-sectional area (Figure 2).

To measure the nasal passage airway volume and minimal cross-sectional area we performed the method first described by Tsolakis et al., in 2016 [38]. More specifically, once the orientation of the skull was performed, a vertical plane was constructed perpendicular to the horizontal border. The vertical plane was then used to rotate the axial slice 90 degrees, making it parallel to the horizontal line. After the skull was properly oriented, borders of the nasal passage are drawn on all three slices: axial, coronal, and sagittal. The nasal passage volume (NP) was defined as the airway volume between the palatal plane and a parallel plane passing through the last axial slice before the nasal septum fused with the posterior pharyngeal wall. This area contains the inferior part of the nasal cavity and the nasopharynx. The minimal cross-sectional area for that volume was also calculated (Figure 2).

### Statistical Analysis

An Excel spreadsheet (Microsoft, Redmond, WA, USA) was used to import all airway volume (V) and minimal cross-sectional area (MCS) measurement data, and SPSS software (IBM, Armonk, NY, USA; version 27) was used to conduct the statistical analysis. For the comparisons of airway volume (mm^3^) measurements in the three-time points, repeated measures ANOVA was used since the normality assumption was validated using the Shapiro–Wilk test. Pairwise post hoc comparisons with Bonferroni correction were used to identify significant changes between time points. For the comparison of minimal cross-sectional area (mm^2^) measurements, Friedman non-parametric test was used since normality assumption was violated, followed by post hoc comparisons with Bonferroni correction. The intraclass correlation coefficient (ICC) was used to assess the intra-method agreement. All tests were 2-sided at α = 5% level of statistical significance.

## 3. Results

The sample included 14 subjects, 6 males and 8 females, with a mean age of 10.82 and 11.34 years, respectively. The operator’s reliability was calculated using intraclass correlation on five randomly selected subjects, whose data were re-measured 2 weeks apart. Every measurement displayed a high intraclass correlation (Table 1).

### 3.1. Airway Volume

**Figure 3:**
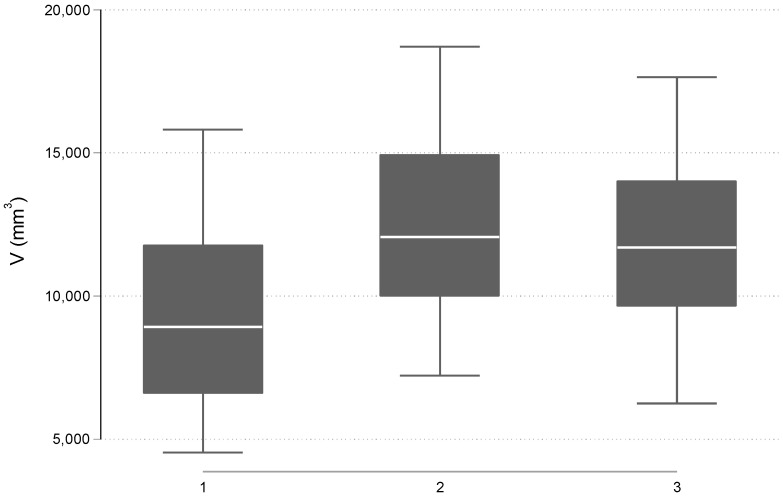
Box plots of the distribution of V (mm^3^) before RPE (time 1), after the end of the expansion (time 2) and 6 months post-expansion (time 3).

According to the measurements of airway volume, the mean value in T1 was 9292.2 ± 3595.0 mm^3^, in T2 was 12,319.1 ± 3274.6 mm^3^, and in T3 was 11,731.3 ± 3260.6 mm^3^. Overall, the airway volumes in the three-time points were statistically significant different with a *p* < 0.001. Particularly, a statistically significant difference was found between the airway volume of T1 and T2 time points with a *p* < 0.001. A statistically significant difference was found by comparing the T1 and T3 airway volumes with a *p* = 0.001. Lastly, when the T2 and T3 airway volumes were compared, the results revealed a statistically significant difference with a *p* < 0.001 (Table 2, Figure 3).

### 3.2. Minimal Cross-Sectional Area

**Figure 4:**
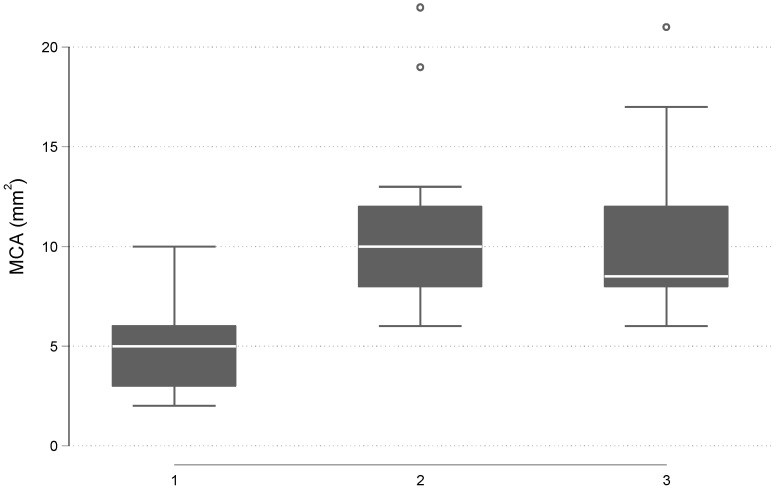
Box plots of the distribution of MCA (mm^2^) before RPE (time 1), after the end of the expansion (time 2), and 6 months post-expansion (time 3).

Regarding the measurements of the airway minimal cross-sectional area, the mean value in T1 was 5.0 ± 2.3 mm^2^, in T2 was 11.0 ± 4.5 mm^2^, and in T3 was 10.3 ± 4.2 mm^2^. Overall, the minimal cross-sectional areas in the three-time points were statistically significantly different with a *p* = 0.001. Particularly, a statistically significant difference was found between the airway minimal cross-sectional area of T1 and T2 time points with a *p* = 0.003. A statistically significant difference was found by comparing the T1 and T3 airway minimal cross-sectional area with a *p* = 0.003. On the contrary, when the T2 and T3 airway minimal cross-sectional areas were compared, the results revealed no statistically significant difference with a *p* = 0.056 (Table 2, Figure 4).

## 4. Discussion

Rapid palatal expansion has been suggested for the treatment of chronic respiratory disorders and, more specifically, obstructive sleep apnea (OSA). The symptoms of obstructive sleep apnea (OSA) include episodes of whole or partial airway collapse, decreased oxygen saturation, and awakening from sleep. Cistulli et al. were one of the first to study the effects of RPE in a sample of ten individuals with mild to moderate OSA [48]. The index score for respiratory distress decreased in all of these patients; nine of these participants’ snoring decreased, and these nine patients experienced less daytime sleepiness. The index for respiratory distress recovered to normal in seven instances. Nine of these participants also experienced less daytime fatigue and snoring. The respiratory distress score decreased in all of these patients. After suffering respiratory distress, seven people’s respiration ratings returned to normal. Rapid palatal expansion may be a beneficial therapy strategy for some OSA patients, according to the authors’ findings [48]. It is really important to mention that nasal airway resistance will be improved by significantly increasing the minimal cross-sectional area and not the entire volume necessary [3,12].

The current prospective clinical investigation used Cone-Beam Computed Tomography (CBCT) images to assess the effect of rapid palatal expansion on the airway volume and minimal cross-sectional area in growing patients treated with a Hyrax appliance. One type of RPE appliance that is tooth-borne is called the Hyrax appliance. The Haas and Hyrax appliances are two distinct tooth-born appliances. The Hyrax device was used for this investigation because it is the most popular RPE appliance and is more hygienic for the patient than the Haas. The Haas device is a fixed split acrylic appliance, and because the acrylic touches the alveolar ridges, there is a chance that the palate mucosa could become inflamed.

While there are a lot of studies in the literature on anatomical changes of the airway after rapid maxillary expansion with Hyrax appliance, there are only a few studies that looked over the changes of nasal passage airway volume and minimal cross-sectional area. In 2014, El H. and Palomo J.M. evaluated retrospectively the nasal passage volume changes that occur after rapid palatal expansion by using a Hyrax expander [36]. Their results revealed that the airway volume of the nasal passage was significantly increased for the patients that underwent rapid palatal expansion treatment. Furthermore, there was no report on the minimal cross-sectional area. In 2020, Lanteri et al. evaluated retrospectively the airway volume in nasal cavity and in the nasopharyngeal area [49]. Their results revealed a statistically increased airway volume in both anatomical areas. The minimal cross-sectional area was not extracted in this study either. Finally, in 2023, Korayem studied retrospectively the airway volume and minimal cross-sectional area changes after rapid palatal expansion [50]. His results suggested that there were no statistically significant differences in airway volume and minimal cross-sectional area between the experimental and control group. It is worth mentioning that current orthodontic literature does not offer solid information about the effect of palatal expansion on the pharyngeal airway, despite the fact that the pharyngeal airway modifications following the rapid palatal expansion are seen clinically [51].

The results of our study suggested that there were statistically significant differences in the airway volume measurements at all time points. More specifically, the airway volume appears to be increased immediately after the expansion is completed. Six months after, the difference between the pre-treatment airway volume is significantly increased, but it seems to be decreased when we compare it to the airway volume immediately after the expansion is completed. This could be happening because in the T2 time point of expansion the airway analysis could possibly include an area of the opened palatal suture that in T3 time point could be bony structure. The minimal cross-sectional area was increased immediately after the expansion was performed, and it remained stable 6 months after the expansion. Those results suggest that palatal expansion could benefit respiratory disorders treatment when the obstruction is located in the nasal passage anatomical area.

The limitation of this study is that we could not add more time points in our study to measure the long-term changes in volume and minimal cross-sectional area. This is because of ethical reasons. Future studies that will look over the volume and minimal cross-sectional area changes for more than 6 months post-treatment time will be needed.

## 5. Conclusions

Rapid palatal expansion can significantly increase the airway volume and minimal cross-sectional area in the nasal passage. The airway volume is significantly increased immediately after expansion and remains increased after the retention period, while it is decreased when the immediately after expansion volume is compared to the post-retention period. The minimal cross-sectional area is significantly increased after expansion and remains equally increased after the retention period.

## Figures and Tables

**Table 1:** Intraclass correlation coefficient (ICC) and 95% confidence interval (CI) for intra-method agreement. Airway volume (V), Minimal cross-sectional area (MSC).

Parameter	ICC (95% CI)
V (mm^3^)	0.85 (0.84, 0.86)
MSC (mm^2^)	0.83 (0.81, 0.86)

**Table 2:** Distribution parameters of V (mm^3^) and MCA (mm^2^) before RPE (time 1), after the end of expansion (time 2) and 6 months post-expansion (time 3). * Bonf. Corrected *p*-values: 1 vs. 2 *p* < 0.001; 1 vs. 3 *p* = 0.001; 2 vs. 3 *p* < 0.001, ** Bonf. Corrected *p*-values: 1 vs. 2 *p* = 0.003; 1 vs. 3 *p* = 0.003; 2 vs. 3 *p* = 0.056.

	Time				
V (mm^3^)	1	2	3	Overall	*p*-Value
Mean (SD)	9292.2 (3595.0)	12,319.1 (3274.6)	11,731.3 (3260.6)	11,114.2 (3553.6)	<0.001 *
Median (IQR)	8922.0 (6607.0, 11,768.0)	12,071.0 (10,001.0, 14,923.0)	11,693.5 (9652.0, 14,012.0)	10,981.5 (8790.0, 13,821.0)	
**MCA (mm^2^)**					
Mean (SD)	5.0 (2.3)	11.0 (4.5)	10.3 (4.2)	8.8 (4.6)	
Median (IQR)	5.0 (3.0, 6.0)	10.0 (8.0, 12.0)	8.5 (8.0, 12.0)	8.0 (6.0, 11.0)	0.001 **

## Data Availability

The data presented in this study are available on request from the corresponding author. The data are not publicly available due to privacy reasons.
